# The effect of partitioned framing vs. all-inclusive framing of donation amount on prosocial behavior: focus on the moderation effect of psychological characteristics

**DOI:** 10.3389/fpsyg.2023.1166092

**Published:** 2023-05-05

**Authors:** Eun Young Lee, Kyounghee Chu

**Affiliations:** ^1^Institute for Business Research & Education, Korea University, Seoul, Republic of Korea; ^2^Division of Business, Chosun University, Gwangju, Republic of Korea

**Keywords:** framing, donation, partitioned framing, all-inclusive framing, need for cognition, regulatory focus, perceived authenticity, prosocial behavior

## Abstract

Despite numerous studies on individual charitable donations and cause-related marketing have been conducted, the framing of the donation amount has not been studied. This research suggests that people's intention to donate to charity differs depending on whether the donation amount is framed as all-inclusive or partitioned. The main effect of partitioned framing was moderated by individual differences in the need for cognition and regulatory focus. The results of our research are threefold. First, people responded more positively to engage in prosocial behavior in the partitioned donation amount condition than in the all-inclusive condition, even when the total amounts were the same. Second, the framing effect of the donation amount differed according to the need for cognition. Individuals with a high need for cognition (NFC) had a higher intention to donate in the partitioned donation amount condition than in the all-inclusive condition, while individuals with low NFC did not show differences in either condition. Third, the framing effect of the donation amount differed according to regulatory focus. Prevention-focused individuals were more willing to donate in the partitioned condition than in the all-inclusive condition, while promotion-focused individuals did not show differences in either condition. In addition, the interaction of framing and regulatory focus on donation intention was mediated by the perceived authenticity of the donation organization. This research has several academic and practical implications for effective corporate social responsibility activities.

## 1. Introduction

As prosocial behaviors such as charitable donations continue to increase, numerous studies related to this topic have been conducted. This research focuses on two specific areas: the circumstances that promote prosocial behavior and the characteristics of individuals who exhibit higher prosocial behavior in specific circumstances. Previous research has explored variables related to circumstances that trigger charitable donation intentions. For instance, the form of donation (money vs. time) (Liu and Arker, 2008), the appeal of the benefit of donation (self-benefit vs. benefit for others) (White and Peloza, 2009), and the message framing of persuasive appeals for donations were found to influence the intention to donate (Olsen et al., 2003; Grau and Garretson, 2007; Grau et al., 2007; Chang and Lee, 2009). In addition, perceptual cues such as changing the context by background color interacted with the frame of the message (gain vs. loss) altering peoples' donation intentions (Kim and Jang, 2018). These findings suggest that merely changing the format or frame of the prosocial campaign information can alter people's thoughts, attitudes, and eventually behavior. For instance, previous studies have shown that message appeals presented in positive and negative frames (e.g., Grewal et al., 1994; Kim and Song, 2023), framing of attribute information of a product (e.g., 75% lean vs. 25% fat) (e.g., Levin and Gaeth, 1988), framing of gain and loss (e.g., Nabi et al., 2020; Gantiva et al., 2021; Gursoy et al., 2022; Kim, 2022; Peng et al., 2022; Stadlthanner et al., 2022; Ort et al., 2023), and presentation of prices (e.g., partitioned price vs. all-inclusive) (e.g., Morwitz et al., 1998; Hamilton and Srivastava, 2008; Völckner et al., 2012; Choi et al., 2020; Marquez et al., 2022; Wang et al., 2023) can yield different results depending on the way the information is presented.

Importantly, in charitable donation literature, research suggests that framing the donation value can alter people's perceptions. For instance, Chang (2008) suggested that framing donation amount in terms of absolute dollar vs. percentage (of a sale price) affects donation differently and that framing the donation information in terms of absolute dollar is more effective when the donation magnitude is low. However, no research has explored whether partitioned framing of the donation amount can change people's prosocial behavior. Specifically, the donation amount can be presented in a single monetary value with details of donation activities, or the same donation amount can be presented in a partition, linking to each donation activity. For instance, if the total donation amount is $12 and the implied donation details or specific activities that the money would be spent on are (1) providing malnutrition treatment for children dying of hunger, $6; (2) aid for refugee children, $3; and (3) education support for children, $3.

This research investigates the impact of framing the donation amount in partitioned vs. all-inclusive form on online prosocial behavior, which has not been studied so far. The study examines how this framing effect is moderated by individual traits, such as the need for cognition and regulatory focus, and how it is mediated by the perceived authenticity of the donation organization. Thus, this research provides innovative insights into the complex interplay between framing, individual differences, and perceived authenticity in shaping people's intentions to donate.

## 2. Literature review

### 2.1. Prosocial behavior in charitable donation and frame of donation information

Previous research suggests that demographic variables such as income (e.g., Auten et al., 2002), marriage (e.g., Brown and Ferris, 2007), religion (e.g., Chang, 2005), and age (e.g., Nichols, 1992) are antecedents of donation intentions. In addition, personal traits such as gender identity, moral identity, altruism, empathy, and regulatory focus have also been found to impact donation intentions (e.g., Andreoni, 1989; Winterich et al., 2009; Verhaert and Van den Poel, 2011; Park and Ryu, 2018; Yen and Yang, 2018). Specifically, individuals with high moral identity and high empathy tend to donate more (Eisenberg and Miller, 1987), but there are also boundary conditions where moral identity decreases donations if the recipients of the donation are perceived as responsible for their woeful plight (Lee S. et al., 2014).

Moreover, the format or frame of donation information has been a key issue in charitable donation research. It has been found that the presentation or framing of the donation information can have a significant impact on people's judgments and decisions (Olsen et al., 2003; Grau et al., 2007). For example, in the area of cause-related marketing, people's perceptions differ based on the format in which the donation amount is presented, such as a percentage of profit vs. a percentage of the price (e.g., 5% of profit vs. 5% of retail price). Similarly, the structure of the donation amount matters, with exact (e.g., 10 cents), calculable (e.g., 5% of price), estimable (e.g., 5% of profit), and abstract (e.g., a portion of the sales) formats having different effects. The exact format was found to be the most trusted donation quantifier (Grau et al., 2007). In the context of marketing, whenever a person makes a purchase, the person would more likely to intend to buy the product when the donation information was framed in terms of absolute dollar vs. percentage as a donation to charity. However, this effect only remained when the donation amount was low; when the donation amount was high, there was no donation framing effect (Chang, 2008). Previous research indicates that not only the personal factors influence people's donation intentions but also the framing of the donation information affect people's perceptions of donation activities.

### 2.2. Partitioned donation amount vs. all-inclusive donation amount

The concept of partitioning the donation amount vs. all-inclusive donation amount originates from research on partitioning prices in consumer behavior. Partitioned pricing refers to dividing the price into two or more components (Morwitz et al., 1998; Völckner et al., 2012). This pricing strategy is perceived to be effective because consumers perceive the partitioned price condition as a smaller amount (less loss) than the all-inclusive price condition with the same total amount. Additionally, the components of partitioned pricing appear to be more salient (more gains) compared to the all-inclusive condition (Chakravarti et al., 2002; Hamilton and Srivastava, 2008). The basic premise is that the partitioned condition enables people to link each component to a monetary value. If the partitioned components, i.e., the donation activities, are salient in the charitable donation context, it would lead the donors to perceive their donation value as larger than that in the all-inclusive condition. In the partitioned donation amount condition, each detail regarding the donation activities is linked to a specific monetary value one by one, making each component (donation activity) more salient and valuable, while the all-inclusive condition presents the donation activities as lumped together.

Furthermore, in the partitioned condition, people will link each donation detail to a separated monetary value, whereas in the all-inclusive condition, the combined details of donation activities would be linked to one monetary value. We hypothesize that if potential donors are exposed to a partitioned donation amount condition when evaluating the charity activity information of an organization, they will attend to all details of the charity activities, including the allocated monetary value of their donation money. In this partitioned presentation condition, the perceived total value of donation money is more likely to appear larger than that in the lump-sum presentation condition. Therefore, in the context of framing the monetary value of the donation, it is predictable that if the donation amount is partitioned, then each donation activity would become more salient and valuable. Thus, the hypothesis 1 suggests the main effect of framing the donation amount (partitioned vs. all-inclusive) as follows:

**Hypothesis 1 (H1)**. Individuals will have a higher intention to donate when the donation amount is partitioned than when the donation amount is all-inclusive.

### 2.3. Need for cognition and partitioned framing

Need for cognition (NFC) is defined as “an individual's tendency to engage in and enjoy effortful cognitive endeavors” (Cacioppo et al., 1984, p. 306). Research has shown that individuals with different levels of NFC tend to react differently in various circumstances. For instance, high NFC individuals are more likely to organize and elaborate on given information (Cohen et al., 1955). They have a natural tendency to seek knowledge and have a desire to control their environment (Verplanken et al., 1992; Thompson et al., 1993). Additionally, high NFC individuals tend to adopt new technology, such as the use of smartphones, more readily than low NFC individuals (Cho and Park, 2014). Previous literature has explained this difference as being due to different information processing mechanisms. While people with high NFC tend to process information systematically and cognitively, those with low NFC tend to process information using cognitive shortcuts (Zhang, 1996).

According to resource-matching theory (Peracchio and Meyers-Levy, 1997), when processing a message, judgments are made more favorably when cognitive resources are not exceeded in comprehending the message. Since high NFC individuals tend to enjoy putting cognitive effort into processing information, messages that require relatively more elaboration would be more appealing to them than to low NFC individuals. Due to the difference in information processing tendencies, we assume that different presentations of information about monetary value will result in different attitudes and intentions toward prosocial behavior for high NFC and low NFC individuals.

**Hypothesis 2-1 (H2-1)**. High need for cognition individuals will have higher intention to donate when the donation amount is partitioned than when the donation amount is all-inclusive.

**Hypothesis 2-2 (H2-2)**. Low need for cognition individuals will not have different donation intentions in partitioned donation amount condition and all-inclusive donation amount condition.

### 2.4. Regulatory focus and partitioned framing

Regulatory focus theory suggests that people's tendencies can be categorized as promotion focus or prevention focus based on their motives (Higgins, 1997, 2002). Promotion-focused individuals place more weight on aspirations, success, and desires, while prevention-focused individuals prioritize duty and responsibility. Promotion-focused individuals typically choose an approach strategy to achieve their goals, while prevention-focused individuals tend to choose an avoidance strategy to prevent undesirable outcomes. As a result, promotion-focused and prevention-focused individuals perceive and behave differently in various situations. Promotion-focused individuals are sensitive to errors of omission or the loss of accomplishments, while prevention-focused individuals are sensitive to errors of commission or committing mistakes. Promotion-focused individuals are more risk-taking, biased, and venturesome, whereas prevention-focused individuals are more conservative, biased, and safety-oriented (Crowe and Higgins, 1997). Promotion-focused individuals are more accommodating in uncertain information as long as it leads to better results, but prevention-focused individuals prefer safe and certain information (Higgins, 2000).

Many research studies have found that regulatory focus plays a moderating role in various contexts, including choice (e.g., Chernev, 2004), decision making (e.g., Crowe and Higgins, 1997; Higgins, 2002), emotional expression (e.g., Higgins et al., 1997), and information processing (e.g., Jain et al., 2006). According to Förster et al. (2003), promotion-focused individuals prioritize speed, while prevention-focused individuals prioritize accuracy when processing information. Promotion-focused individuals place more value on broadly framed information, while prevention-focused individuals value detailed information more and considering it safer. Prevention-focused individuals tend to process information locally, narrowly, and in detail (Lee K. et al., 2014). Given these differences in information processing, we suggest that promotion-focused and prevention-focused individuals will react differently to the framing of the donation amounts. Specifically, although the total donation amount is equivalent, the partitioned condition presents details of the charity activities and the allocation of the donation money. Since prevention-focused individuals prefer safer and more detailed information over uncertain and risky alternatives, partitioning the donation amount will be preferred over the all-inclusive donation amount. Thus, the suggested hypotheses are as follows:

**Hypothesis 3-1 (H3-1)**. Prevention-focused individuals will have a higher intention to donate when the donation amount is partitioned than when the donation amount is all-inclusive.

**Hypothesis 3-2 (H3-2)**. Promotion-focused individuals will not have different donation intentions in partitioned donation amount condition and all-inclusive donation amount condition.

Furthermore, the relationship between the interaction of framing the donation amount and regulatory focus on donation intention will be mediated by people's perception of authenticity and sincerity toward the donation organization. Authenticity generally refers to being true to oneself (individual, corporate, organization, etc.) and having actions and activities that are in accordance with one's values (Gardner et al., 2005; Mazutis and Slawinski, 2015). In addition, in Corporate Social Responsibility (CSR) literature, authenticity is referred to as whether a firm's CSR efforts are genuine (Mazutis and Slawinski, 2015). Sincerity refers to being honest and trustworthy. Wicki and Van Der Kaaij (2007) found that if an organization is perceived as authentic, the stakeholders are more likely to trust the CSR efforts of the organization. People's perceptions of the organization are very important since perception is a key factor in raising money for donation activities.

If the organization reveals more details and provides information about the allocation of the monetary value, people would regard the organization as sincere, transparent, or honest. Additionally, the donation organization, which is providing information that is linked separately with each monetary value would make people think that the charity organization is providing more information about each donation activity, thus leading them to perceive the organization to be authentic and sincere. Therefore, we hypothesize that the effect of the interaction of framing the donation amount and regulatory focus on donation intention will be mediated by people's perceptions of authenticity, sincerity, or trust toward the organization. Hypothesis 4 is as follows:

**Hypothesis 4 (H4)**. The interaction of framing donation amount and regulatory focus will be mediated by perceived authenticity toward the organization.

**Figure 1:**
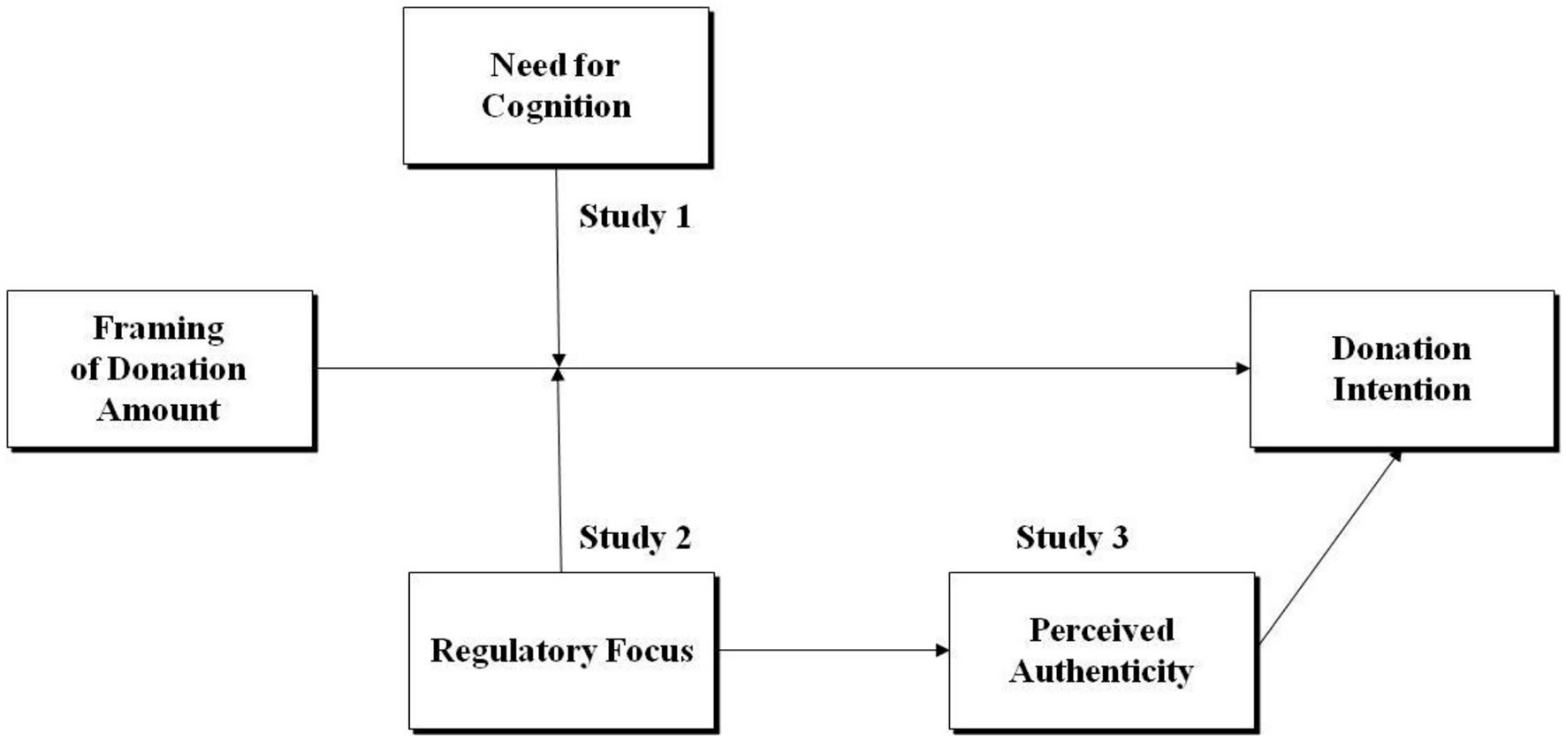
Conceptual framework.

Figure 1 illustrates the conceptual framework of our study. It suggests that the framing of the donation amount in a prosocial campaign, which would influence people's donation intention. This main effect would be moderated by two variables: the first being Need for Cognition (Study 1), and the second being Regulatory Focus (Study 2). Additionally, the framework indicates that the interaction between the framing of the donation amount and regulatory focus would be mediated by perceived authenticity, which in turn would affect donation intention.

## 3. Study 1

### 3.1. Materials and methods

#### 3.1.1. Data collection and procedures

The purpose of Study 1 was to examine the effect of framing donation amounts as partitioned vs. all-inclusive and to test the moderation of NFC (need for cognition). Figure 1 presents the conceptual framework of this study. The experimental design for Study 1 was a between-subjects design with two factors: framing of donation amount (partitioned vs. all-inclusive) and need for cognition (low NFC vs. high NFC). The participants were randomly assigned to either the partitioned condition or to the all-inclusive condition. Their need for cognition was measured and the respondents were sorted into low and high NFC groups.

The data were collected from university students in South Korea, who participated in the study in exchange for course credit. The study was conducted online, without face-to-face interaction. Participants could access the study by clicking on a survey URL link. Before beginning the online survey, all participants provided their consent to participate in the study. The survey did not ask for any personal or sensitive information, and the participants were notified that all collected data would be used solely for academic research purposes. A total of 151 respondents were included in the data, of which 70 were males (46.4%) and 81 were females (53.6%). Their age range of the participants was between 20 and 30 years.

The survey procedure was as follows: First, the participants were asked to review an advertisement for a prosocial campaign that focused on “support for children who need help.” In the partitioned condition, the specific donation amounts for different causes were given, with “Malnutrition treatment, $6,” “Aid for refugee children, $3,” “Education support for children, $3,” and a total of $12. On the contrary, in the all-inclusive condition, the message was presented as “Malnutrition treatment, aid for refugee children, education support for children, $12.” The respondents were exposed to the campaign advertisement for at least 15 s. Then, the respondents were presented with a manipulation check questionnaire, a donation intention questionnaire, an attitude toward the organization questionnaire, questions about their psychological characteristics, and demographic variables.

Before getting into the main study, we conducted a pretest to determine the fair amount of money that university students would be willing to donate to non-profit organizations. We asked an open-ended question and received responses ranging from 10,000 won to 16,000 won (equivalent to $8 to $12.31), and we determined that $12 would be a fair amount to donate. We also decided on the partitioned donation amounts (i.e., $3, $3, and $6) through a pretest, as they were well balanced.

#### 3.1.2. Measurement variables

The measurement item for donation intention was “I intend to donate to this organization if I have the opportunity” (1 = “not at all”; 7 = “very much”). The measurement of the need for cognition was based on the items from Caccioppo and Petty (1982, 1984) and Lins de Holanda Coelho et al. (2020). The items in the questionnaire were “I prefer complex problems to simple ones,” “I enjoy taking responsibility for situations that require a lot of thinking,” “Thinking is not my idea of fun (R),” “I would rather do something that requires little thought than something that is sure to challenge my thinking abilities (R),” and “I really enjoy a task that involves coming up with new solutions to problems,” scored on a 7-point scale from 1 (“'not at all”) to 7 (“very much”). The Cronbach's α for the NFC scale was 0.843, indicating high reliability. To distinguish between high NFC and low NFC, the median was used as a cutoff point, as in Caccioppo et al. (1982, 1984). Scores higher than the median were classified as “High NFC,” while scores lower than the median were classified as “Low NFC.”

### 3.2. Results

A two-way ANOVA test is performed to test the hypothesis (Table 1). The dependent variable was donation intention, and a significant main effect was found. Respondents showed a higher donation intention in the partitioned donation amount condition than in the all-inclusive donation amount condition [*M*_partitioned_ = 3.28, standard deviation (SD) = 1.50, *M*_all − inclusive_ = 2.68, SD = 1.23, *F*_(1, 147)_ = 7.14, *p* = 0.008]. Therefore, H1 is supported by the results.

**Table 1:** Two-way ANOVA test results of Study 1.

	**SS**	**d.f**.	**MS**	**F-value**	**Sig**.
The frame of donation amount (1)	13.316	1	13.316	7.144	0.008
NFC (2)	1.727	1	1.727	0.927	0.337
(1) × (2)	4.297	1	4.297	2.306	0.131
Error	273.985	147	1.864		

**Figure 2:**
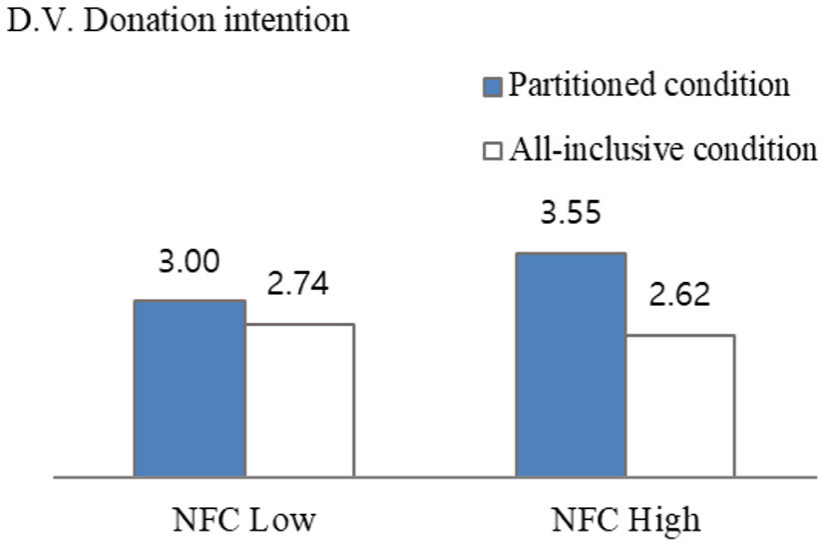
Interaction effect of framing donation amount and NFC (Study 1).

To test whether donation intention differed according to the need for cognition, planned contrast was performed. For high NFC, the difference between partitioned condition vs. all-inclusive condition was significant [*M*_partitioned_ = 3.55 vs. *M*_all − inclusive_ = 2.62, *F*_(1, 147)_ = 9.156, *p* = 0.000]. However, for low NFC, the difference between partitioned condition vs. all-inclusive condition was insignificant [*M*_partitioned_ = 3.00 vs. *M*_all − inclusive_ = 2.74, *F*_(1, 147)_ = 0.618, *p* > 0.1; Figure 2]. Thus the results supported H2-1 and H2-2.

## 4. Study 2

### 4.1. Materials and methods

#### 4.1.1. Data collection and procedures

The purpose of Study 2 was to verify the main effect of partitioned framing with a different stimulus and to test the moderating effect of regulatory focus. The data for Study 2 were collected through Amazon Mechanical Turk (MTurk), an online survey platform that has a pool of participants who can be recruited to take part in online surveys. The participants who took part in the survey were compensated with some amount of payment. The study was conducted online, without face-to-face interaction. Participants could access the study by clicking on a survey URL link. Before beginning the online survey, all participants provided their consent to participate in the study. The survey did not ask for any personal or sensitive information of the participants, and they were notified that all collected data would be used solely for academic research purposes. The data included 247 replies, with 100 female respondents (40.5%), and 147 male respondents (59.5%). The age distribution of participants was as follows: between 20 and 30 years (38.9%), between 30 and 40 years (35.2%), between 40 and 50 years (13.4%), and between 50 and 60 years (12.5%).

The study utilized a 2 (framing of donation the amount: partitioned framing vs. all-inclusive framing) × 2 (regulatory focus: promotion focus vs. prevention focus) between-subjects design. The participants were randomly assigned to either the partitioned condition or to the all-inclusive condition. The regulatory focus of the participants was measured, and the participants were arranged into either promotion-focused or prevention-focused groups based on the median value.

The survey procedures were as follows: First, participants were asked to review an online prosocial donation campaign advertisement. The message for both conditions was “A Little Help for Disaster Recovery Does Make a Difference!” In the partitioned framing condition, participants were presented with information as follows: “California wildfire. $4, Hurricane/typhoon, $5, Earthquake, $3, Total donation amount, $12.” Conversely, in the all-inclusive framing condition, participants were given information as follows: “Donation amount: $12, Donation details: California wildfire, Hurricane/typhoon, and Earthquake”. After viewing the campaign messages for at least 15 s, participants completed a questionnaire regarding their donation intention, evaluation of the organization, personal tendencies, and other related factors.

#### 4.1.2. Measurement variables

The measurement for donation intention was identical with Study 1 [“I intend to donate to this organization if I have a chance” (1 = “not at all”; 7 = “very much”)]. For regulatory focus, the items were adapted from Haws et al. (2010). The items were as follows: (1) When it comes to achieving things that are important to me, I find that I don't perform as well as I would ideally like to do (reverse code), (2) I usually obeyed rules and regulations that were established by my parents, (3) I feel like I have made progress toward being successful in my life, (4) Not being careful enough has gotten me into trouble at times (reverse code), (5) When I see an opportunity for something I like, I get excited right away, (6) I worry about making mistakes, (7) I frequently imagine how I will achieve my hopes and aspirations, (8) I frequently think about how I can prevent failures in my life, (9) I see myself as someone who is primarily striving to reach my “ideal self”—to fulfill my hopes, wishes, and aspirations, and (10) I see myself as someone who is primarily striving to become the self I “ought” to be—fulfill my duties, responsibilities, and obligations. Among the questionnaire, statements (1), (3), (5), (7), and (9) were related to promotion focus, and statements (2), (4), and (6), (8), (10) were related to prevention focus. To assess the reliability of the scale, we conducted a reliability analysis and obtained Cronbach's α coefficients of 0.793 and 0.704 for each factor, respectively, indicating acceptable levels of reliability for the scale. After averaging the promotion and prevention focus scores, we calculated the difference between the two scores. Then, we performed a median split, where a score higher than the median was classified as promotion focus, and a score lower than the median was classified as prevention focus.

### 4.2. Results

**Figure 3:**
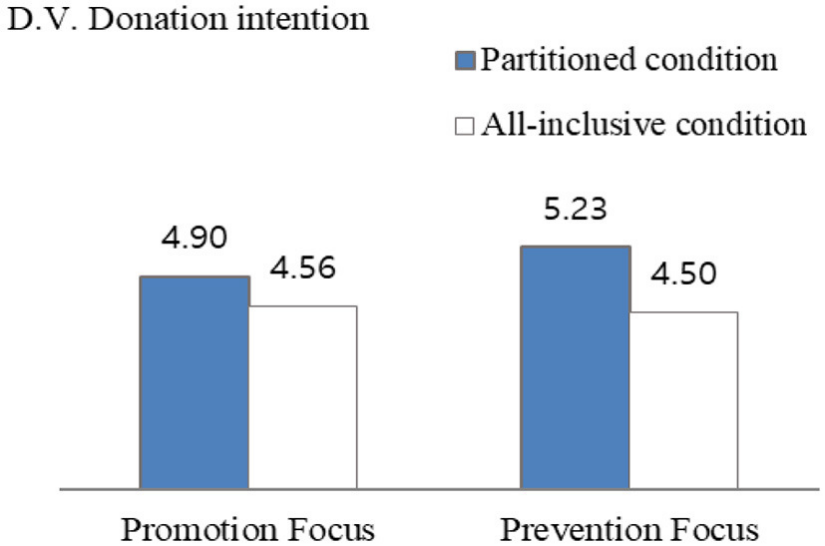
Interaction effect of framing donation amount and regulatory focus on donation intention (Study 2).

Table 2 shows the summary of a two-way ANOVA experiment that is performed to test the hypotheses. The dependent variable was donation intention and the main effect of partitioned framing was found. Respondents revealed higher donation intention in the partitioned donation amount condition than in the all-inclusive donation amount condition [*M*_partitioned_ = 5.09, SD = 1.54, *M*_all − inclusive_ = 4.53, SD = 1.86, *F*_(1, 243)_ = 5.96, *p* = 0.015]. Planned-contrast analyses of the specific conditions were performed. For prevention focus, the difference between the partitioned condition vs. all-inclusive condition was significant [*M*_partitioned_ = 5.23 vs. *M*_all − inclusive_ = 4.50, *F*_(1, 243)_ = 6.33, *p* = 0.013]. However, for promotion focus, the difference between partitioned condition vs. all-inclusive condition was insignificant [*M*_partitioned_ = 4.90 vs. *M*_all − inclusive_ = 4.56, *F*_(1, 243)_ = 1.15, *p* > 0.1; see Figure 3]. Thus, the results supported H3-1 and H3-2.

**Table 2:** Two-way ANOVA results of Study 2.

	**SS**	**d.f**.	**MS**	**F-value**	**Sig**.
Frame of donation amount (1)	17.026	1	17.026	5.96	0.015
Regulatory focus (2)	1.066	1	1.066	0.37	0.542
(1) × (2)	2.331	1	2.331	0.82	0.367
Error	694.316	243	2.857		

## 5. Study 3

### 5.1. Materials and methods

#### 5.1.1. Data collection and procedures

The purpose of Study 3 was to support the results of Study 2 and to explore the underlying psychological mechanism of the interaction effect. While the stimulus for Study 2 was a charitable advertisement regarding “help for disaster recovery,” the stimulus for Study 3 was “support for children who need help,” which is identical to the stimulus used in Study 1. The data were gathered from South Korean university students who participated in the study in exchange for academic credit. The study was conducted online, with no face-to-face interaction. Participants were able to access the study by clicking on a survey URL link. Prior to starting the survey, all participants gave their consent to participate, and the survey did not ask for any personal or sensitive information of the participants. Participants were informed that all data collected would be used solely for academic research purposes. A total of 161 respondents were included in the data, among them 89 were females (55.3%) and 72 were males (44.7%). Their age range was between 20 and 30 years.

The study utilized a between-subjects design with two factors: frame (partitioned donation amount vs. all-inclusive donation amount) and regulatory focus (promotion focus vs. prevention focus). The participants were randomly assigned to either the partitioned condition or the all-inclusive condition. The regulatory focus of the participants was measured, and they were sorted into promotion-focused and prevention-focused groups based on the median value.

The experimental procedures followed in this study were as follows: First, participants were asked to review a charitable advertisement. After viewing the donation messages for at least 15 s, participants completed a questionnaire regarding their donation intention, evaluation of the organization, personal tendencies, and other related factors. The items in the regulatory focus questionnaire were identical to those used in Study 2. The Cronbach's α coefficients for the promotion focus and prevention focus scales were 0.701 and 0.602, respectively, indicating acceptable levels of reliability for both scales. These results align with the recommendation by Hair et al. (2010) that a Cronbach's α value above 0.6 can be considered satisfactory for assessing reliability. The perceived authenticity questionnaire used a 7-point scale with the statement “It seems that the donation organization is sincere (1 = ‘not at all'; 7 = ‘very much').”

### 5.2. Results

**Figure 4:**
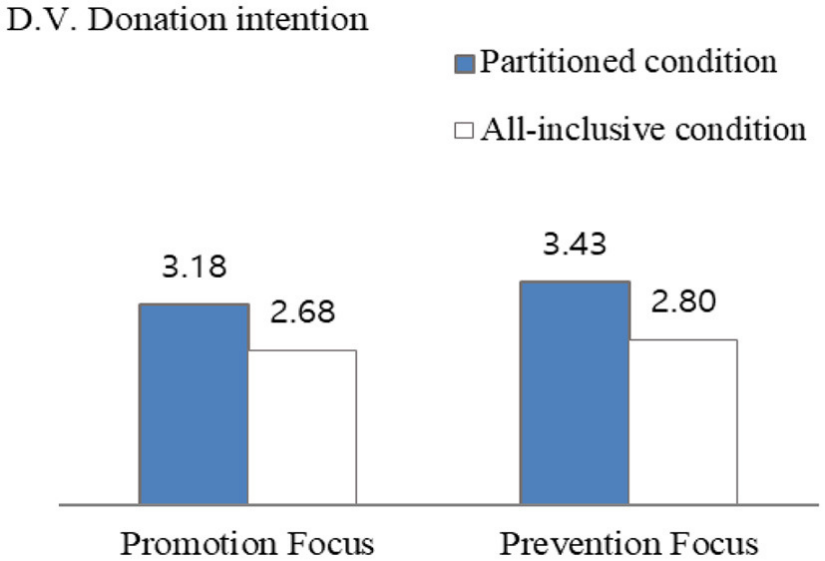
Interaction effect of framing donation amount and regulatory focus on donation intention (Study 3).

As in Table 3, a two-way ANOVA test was performed to test the hypothesis. The dependent variable was donation intention and a significant main effect was found. Respondents showed higher donation intention in the partitioned donation amount condition than in the all-inclusive donation amount condition [*M*_partitioned_ = 3.30, SD = 1.52, *M*_all − inclusive_ = 2.74, SD = 1.32, *F*_(1, 157)_ = 6.14, *p* = 0.014]. Therefore, the results were consistent with Studies 1 and 2. To retest H3, planned contrast analyses of the specific conditions were performed. For prevention focus, the difference between partitioned condition vs. all-inclusive condition was significant [*M*_partitioned_ = 3.43 vs. *M*_all − inclusive_ = 2.80, *F*_(1, 157)_ = 4.23, *p* = 0.041]. However, for promotion focus, the difference between partitioned condition vs. all-inclusive condition was insignificant [*M*_partitioned_ = 3.18 vs. *M*_all − inclusive_ = 2.68, *F*_(1, 157)_ = 2.40, *p* > 0.1; see Figure 4]. Thus, the results supported H3-1 and H3-2, which was consistent with the results of Study 2.

**Table 3:** Two-way ANOVA results of Study 3.

	**SS**	**d.f**.	**MS**	**F-value**	**Sig**.
Frame of donation amount (1)	12.555	1	12.555	6.144	0.014
Regulatory focus (2)	1.356	**1**	1.356	0.663	0.417
(1) × (2)	0.178	1	0.178	0.087	0.768
Error	320.828	157	2.043		

**Figure 5:**
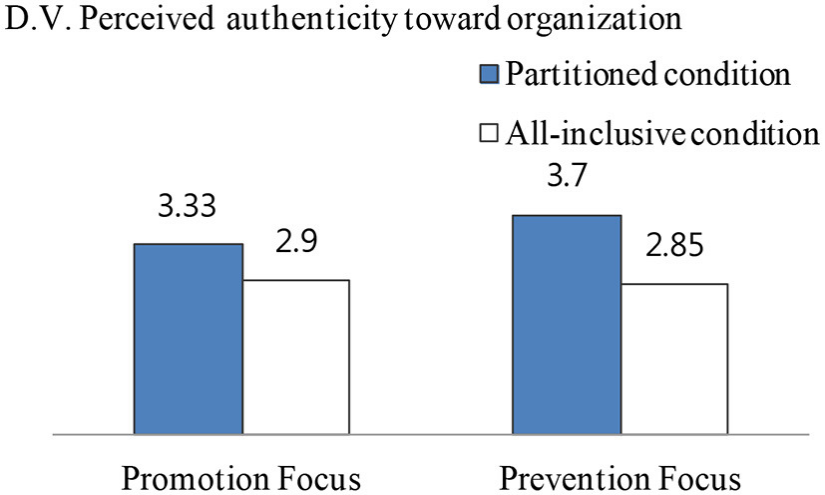
Interaction effect of framing donation amount and regulatory focus on perceived authenticity (Study 3).

**Figure 6:**
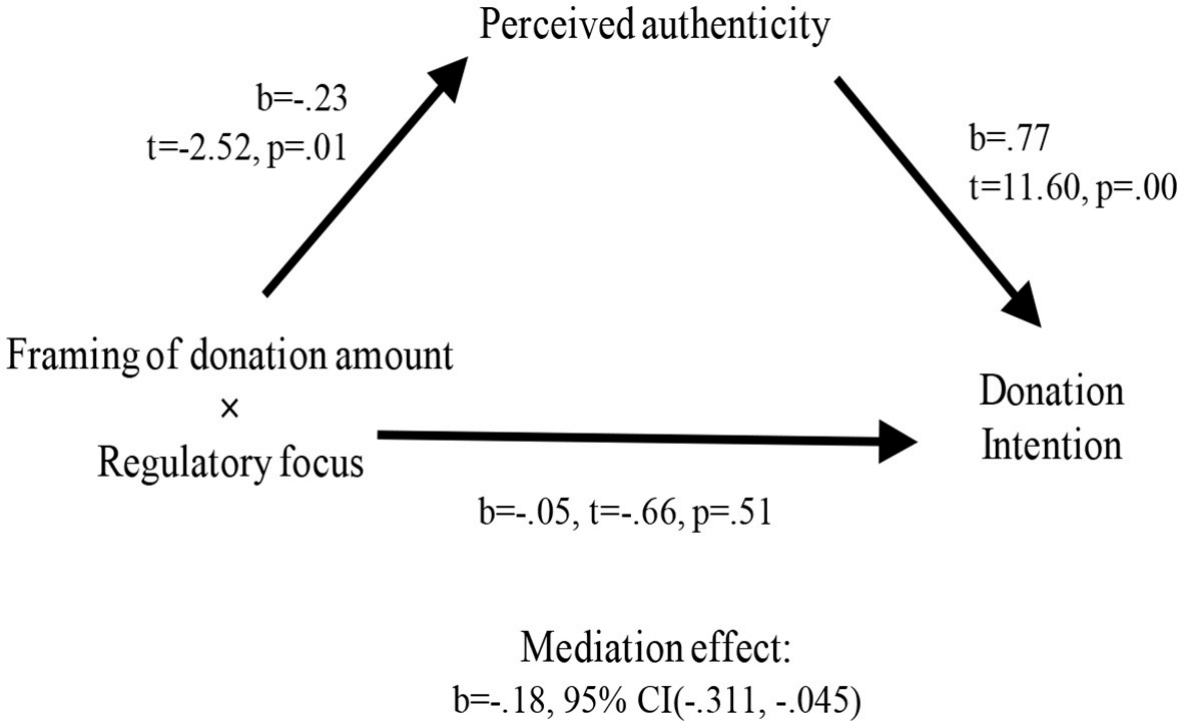
Mediation analysis (Study 3).

Next, using perceived authenticity toward the organization as the dependent variable, the result was consistent with donation intention as the dependent variable. For prevention focus, perceived authenticity toward the organization of partitioned condition was significantly higher than all-inclusive condition [*M*_partitioned_ = 3.70 vs. *M*_all − inclusive_ = 2.85, *F*_(1, 157)_ = 9.32, *p* = 0.003]. For promotion focus, perceived authenticity did not differ significantly between partitioned and all-inclusive conditions [*M*_partitioned_ = 3.33 vs. *M*_all − inclusive_ = 2.90, *F*_(1, 157)_ = 2.33, *p* > 0.1; see Figure 5]. To find out whether perceived authenticity toward the organization mediated the relationship between the interaction of the donation amount frame and regulatory focus with donation intention, mediation analysis is performed. This was done by following the method of Preacher et al. (2007), bootstrapped with the Macro process (Model 4). As a result, perceived authenticity toward the organization mediated the relationship between the interaction of the donation amount frame and regulatory focus and donation intentions. Specifically, the main effect of the interaction of donation amount frame and regulatory focus on the authenticity toward the organization' turned out to be significant (*b* = −0.23; *t* = −2.52; *p* = 0.01). The main effect of authenticity toward the organization on donation intention was significant (*b* = 0.77; *t* = 11.60; *p* = 0.000). The direct effect of the interaction of donation amount's frame and regulatory focus on donation intention was insignificant (*b* = −0.05, *t* = −0.66, *p* = 0.51). Finally, the confidence interval (CI) in the indirect effect of *X* (interaction of donation amount frame and regulatory focus) on *Y* (donation intentions) does not include zero (−0.311, −0.045). Therefore, hypothesis 4, which predicted that authenticity toward the organization, would mediate the relationship between the interaction of donation amount frame and regulatory focus and donation intention was supported (see Figure 6).

## 6. Conclusion

### 6.1. Summary

This research investigates the relationship between the framing of the donation amount and individual differences, specifically the need for cognition and regulatory focus, on prosocial behavior. From the results of the three studies, several findings can be drawn. First, a main effect of the framing of the donation amount was found, with people revealing a higher intention to donate when exposed to the advertisement with a partitioned donation amount compared to an equivalent donation amount presented in an all-inclusive format. Second, the moderating role of the need for cognition was identified, with high NFC individuals revealing a higher intention to donate in partitioned framing compared to all-inclusive framing of the donation amount. Third, the moderating role of regulatory focus was found, with a promotion focus not revealing a significant difference between partitioned and all-inclusive framing of the donation amount, but prevention focus revealing a higher donation intention in partitioned framing than all-inclusive framing of the donation amount. Fourth, the effect was mediated by people's perceived authenticity toward the donation organization. Perceived authenticity toward the donation organization played a mediating role in the relationship between the interaction of framing the donation amount (partitioned vs. all-inclusive) and regulatory focus. Finally, the effect was supported by various respondents through Amazon MTurk (including respondents from English-speaking countries worldwide) and university students in South Korea. Furthermore, the result was consistently supported in different donation categories: Studies 1 and 3 were about donations for disaster recovery, and Study 2 was about donations for children in need of help.

### 6.2. Contributions

This research makes significant contributions to the academic literature in several ways. First, the study enriches the field of individual charitable donations by demonstrating that people respond differently to subtle variations in the framing of the donation amounts. Previous research has primarily focused on altering the valence frame of donation information, peripheral elements, or the message frame, whereas this study is the first to consider the effect of partitioned vs. all-inclusive framing of the donation amount itself. Second, the study identifies the boundary conditions of the main effect of the partitioned framing in the donation amount, illustrating that the effect of partitioned framing varies according to individual differences in need for cognition and regulatory focus. Next, the study examines the psychological mechanism underlying the interaction between framing the donation amount and regulatory focus, revealing that prevention-focused individuals in the partitioned donation amount condition are more willing to donate because they perceive the non-profit organization to be authentic and sincere.

Furthermore, this study proposes a new research direction by linking the partitioned framing effect to prosocial behavior. Specifically, the findings suggest that partitioned framing can influence people's perceptions of the authenticity and sincerity of the non-profit organization, leading to higher donation intentions. This innovative insight opens up new avenues for research exploring the interplay between framing effects and prosocial behavior, highlighting the need for further investigation into other underlying psychological mechanisms such as the higher perceived value of donation amount. By shedding light on this important topic, this study contributes to the advancement of knowledge in the field of charitable donations and provides valuable directions for the future research.

This study provides practical insights into effective communication strategies for charitable organizations to maximize their donation impact and promote online prosocial behavior among young generations. Specifically, the study highlights the importance of partitioning the donation amount into detailed and divided formats, which can increase people's perceptions of the organization's authenticity and lead to higher donation intentions. However, the effectiveness of partition framing varies depending on individual differences in the need for cognition and regulatory focus, emphasizing the need to tailor persuasive messages to the cognitive style and motivational orientation of the audience. Moreover, the study emphasizes the importance of perceived authenticity in online charitable campaigns and highlights the need for non-profit organizations to prioritize building trust with their target audience, taking into account their cognitive and motivational characteristics and utilizing the results of the study on partition framing of the donation amount to customize their messages and donation structure to encourage online prosocial behavior. In addition, the findings of this research can also be applied to various types of online prosocial behavior beyond charitable donations. By considering individual differences in cognitive and motivational factors and tailoring persuasive messages accordingly, organizations and individuals can effectively promote various forms of online prosocial behavior.

### 6.3. Limitations and future research

Despite the valuable contributions of this research, there are several limitations to be acknowledged. Firstly, the sample used in Study 1 and 3 consisted of university students in South Korea, which may limit the generalizability of the results to a broader population and cultures. Therefore, the future studies could replicate the findings using a more diverse sample, including individuals from different age groups and cultural backgrounds. Furthermore, comparative studies across different countries could shed light on potential cross-national differences in donation behavior. These avenues for the future research could enhance the external validity and applicability of the findings to a wider range of populations and contexts.

Secondly, it should be noted that the donation amount used in all studies was fixed at $12 based on the pretest results. While this amount may have been appropriate for the context of the studies, using larger monetary values in the future research could increase the external validity of the findings and provide greater insight into the effects of partitioned framing on donations at different levels of magnitude. Additionally, it would be beneficial for the future research to investigate whether there are differential effects of partitioned framing on donation behavior across varying levels of total donation amount (small, medium, large) and conduct comparative analyses.

Furthermore, it should be acknowledged that this study relied on self-reported measures of donation intentions, which may not fully capture actual behavior. In the future research, it would be valuable to incorporate actual donation amounts as a behavioral measure to enhance the external validity of the results. This would provide a more accurate representation of the effects of partitioned framing on donation behavior and allow for a more comprehensive understanding of the impact of partitioned framing on prosocial behavior. In addition, it may be valuable for the future research to consider utilizing actual organization logos in the stimuli as this may increase the external validity of the study. Incorporating such stimuli may provide a more realistic representation of donation scenarios and could result in increased engagement and investment from participants.

For the future research, exploring additional moderating variables could provide a more nuanced understanding of the effects of partitioned framing. For example, one possibility could be investigating the influence of individual differences in emotional intelligence (e.g., empathy) on the effectiveness of partitioned framing. Another potential variable could be the role of cultural values (individualism–collectivism) in shaping donation behavior. Furthermore, it may be worthwhile to examine whether the effect of partitioning differs for individuals with low levels of trust or cynicism toward charitable organizations. For instance, low-trust individuals may not respond strongly to partitioned framing if they are skeptical about the authenticity of the organization.

Furthermore, despite the acceptable Cronbach's α coefficient for the prevention focus scale in this study (α = 0.602), efforts should be made to increase the scale's reliability in the future research. For instance, while this study measured participants' chronic prevention focus, the future research could use priming methodology to induce prevention focus in a situational context and examine how individuals respond to partitioned framing under these conditions. Such an approach may provide valuable insights into the interaction between regulatory focus and framing effects on charitable donations.

## Data availability statement

The raw data supporting the conclusions of this article will be made available by the authors, without undue reservation.

## Ethics statement

Ethical review and approval was not required for the study of human participants in accordance with the local legislation and institutional requirements. Written informed consent from the patients/participants or patients/participants legal guardian/next of kin was not required to participate in this study in accordance with the national legislation and the institutional requirements.

## Author contributions

EL conceived and proposed the initial research idea, conducted the literature review, and designed and carried out the survey. KC developed the research model and hypotheses, analyzed the data, and revised the manuscript. All authors contributed to the article, reviewed, and approved the final manuscript.
